# The severity of mobile phone addiction and its relationship with quality of life in Chinese university students

**DOI:** 10.7717/peerj.8859

**Published:** 2020-06-01

**Authors:** Lu Li, Grace K.I. Lok, Song Li Mei, Xi Ling Cui, Lin Li, Chee H. Ng, Gabor S. Ungvari, Juan Zhang, Feng Rong An, Yu Tao Xiang

**Affiliations:** 1 The Affiliated Brain Hospital of Guangzhou Medical University (Guangzhou Huiai Hospital), Guangzhou, China; 2 Center for Cognition and Brain Sciences, University of Macau, Macao SAR, China; 3 Kiang Wu Nursing College of Macau, Macao SAR, China; 4 School of Public Health, Jilin University, Changchun, China; 5 Department of Business Administration, Hong Kong Shue Yan University, Hong Kong SAR, China; 6 Department of Pharmacy, The First Affiliated Hospital, College of Medicine, Zhejiang University, Hangzhou, China; 7 Department of Psychiatry, The Melbourne Clinic and St Vincent’s Hospital, University of Melbourne, Richmond, Victoria, Australia; 8 University of Notre Dame Australia, Fremantle, WA, Australia; 9 Division of Psychiatry, School of Medicine, University of Western Australia, Perth, WA, Australia; 10 Faculty of Education, University of Macau, Macao SAR, China; 11 The National Clinical Research Center for Mental Disorders & Beijing Key Laboratory of Mental Disorders, Beijing, China; 12 Beijing Anding Hospital & The Advanced Innovation Center for Human Brain Protection, Beijing, China

**Keywords:** Mobile phone use, Quality of life, University students, China

## Abstract

**Objective:**

This study examined the severity of mobile phone addiction and its relationship with quality of life (QOL) in Chinese university students.

**Methods:**

A total of 2,312 university students from Macao, Hong Kong and mainland China participated in this cross-sectional study. The Mobile Phone Addiction Scale (MPAS), and the World Health Organization Quality of Life-Brief version (WHOQOL-BREF) rating instruments were used to assess the severity of mobile phone addiction and QOL, respectively.

**Results:**

Compared to students in mainland China, those in Macao and Hong Kong were more likely to have excessive mobile phone use. Multiple linear regression revealed that high academic pressure and poor academic performance were positively associated, while male gender, greater interest in academic major and long sleep duration were negatively associated with the severity of mobile phone addiction. Students addicted to mobile phone use had significantly lower scores across all QOL domains.

**Conclusion:**

Due to the adverse impact of excessive mobile phone use on QOL, public education and effective preventive measures should be developed for Chinese university students.

## Introduction

The use of smartphone has exponentially increased in recent years. In 2018, the number of smartphone users has exceeded 2.5 billion people worldwide, while the corresponding figure in China reached around 713 million (Statista Research Department, 2019). Because of their availability, relatively cheap price, and convenience in use, smartphones are quickly replacing laptop and desk-top computers as the preferred method of accessing the internet. Although smartphones have a wide range of functionality that enhances productivity, excessive mobile phone use could lead to negative health outcomes, such as poor sleep quality, headache, tiredness (Zarghami et al., 2015), wrist and neck pain, blurred vision (Rai et al., 2016), sleep quality (Sahin et al., 2013), anxiety, insomnia (Jenaro et al., 2009), and depression (Thomee, Harenstam & Hagberg, 2011), all of which could lead to low quality of life (QOL).

Excessive mobile phone use increases the risk of mobile phone addiction, which is characterized by typical symptoms of addiction (Billieux et al., 2015). The diagnostic criteria of mobile phone addiction remains controversial. Other terms are also used to describe this construct, including addiction of mobile phone use, excessive mobile phone use, compulsive mobile phone use, problematic mobile phone use, pathological mobile phone use, and compensatory mobile phone use (De-Sola Gutierrez, Rodriguez De Fonseca & Rubio, 2016). Mobile phone addiction and Internet addiction share similar features. Mobile phone addiction is occasionally described using the criteria of internet addiction, as both of them are characterized by dependance, tolerance, withdrawal symptoms, and social problems (Beard, 2005).

The China Internet Network Information Center (CINIC) has reported that there were 854 million internet users in mainland China in 2019; of them, 99.1% accessed the Internet via mobile phones, and people aged 20–29 years accounted for the highest proportion of internet users (24.6%) (The China Internet Network Information Centre, 2019). Chinese university students on average spend over 5 h a day on mobile phone and around 79% of students use their mobile phones during class (MyCOS Research Institute, 2018).

There is a lack of research on the association of the severity of mobile phone addiction and QOL in Chinese university students, which gave us the impetus to examine the demographic characteristics and QOL of Chinese university students with excessive mobile phone use. This study also included participants from two special administrative regions of China, the former European colonies of Macau and Hong Kong that have different socioeconomic background compared to mainland China (Wang et al., 2018; Xiang et al., 2008). We hypothesized that certain demographic characteristics would influence the severity of mobile phone use, and more excessive mobile phone use would be associated with poorer QOL in Chinese students.

## Materials and Methods

### Study setting and participants

This study was conducted in one comprehensive university and one nursing college each in Macao and mainland China and one comprehensive university in Hong Kong in 2016. According to the total number of students in each school, 1–5 residential colleges or classes at each study site were randomly selected by computer-generated random numbers. Students in the selected colleges or classes were invited to participate in this survey to complete the rating instruments on demographics, mobile phone use and QOL anonymously within a week. Inclusion criteria were: (1) age 18 years and above. (2) Fluency in Chinese language (Cantonese or Mandarin). (3) Willingness to provide written informed consent. The protocol of the survey has been approved by Clinical Research Ethics Committee of the University of Macao (Approval number: BSERE-APP002-FHS). All participants provided written informed consent.

### Assessment instruments and evaluation

Basic demographic characteristics were collected with a data collection sheet designed for this study. The Mobile Phone Addiction Scale (MPAS) (Hong, Chiu & Huang, 2012) derived from the Young’s Internet Addiction Test (Young, 1998) was applied to rate the severity of mobile phone addiction. The MPAS consists of 11 items, covering three domains of mobile phone use: (1) Time Management (5 items) (2) Academic Issues in School (3 items) (3) Reality Substitute (3 items). Each item was rated from 1 = “do not agree” to 6 = “completely agree”. A higher score indicates more excessive mobile phone use. The MPAS has good psychometric properties established in Chinese college students; its Cronbach’s α is 0.86, while the corresponding figures are 0.83, 0.84, and 0.67 in the three domains, respectively (Hong, Chiu & Huang, 2012). QOL was evaluated with the World Health Organization Quality of Life—brief version (WHOQOL-BREF) (Fang, Hao & Li, 1999; Group, 1997), which is the most widely used QOL measure in Chinese populations with satisfactory reliability and validity (Xia et al., 2012; Zhang et al., 2012). The WHOQOL-BREF consists of 26 items covering physical, psychological, social and environmental domains. A higher score indicates better QOL.

### Statistical analyses

Data analyses were carried out with SPSS 21.0 for Windows statistical package. Normal distribution of continuous variables was measured using the *P*–*P* test. The associations between MPAS total score and related factors was examined with independent samples *t* test, analysis of variance (ANOVA) and Pearson correlation analysis, as appropriate. The independent associations between the severity of mobile phone addiction and QOL were determined with partial correlation analyses controlling for significant demographic correlates of the severity of mobile phone addiction found in the univariate analyses. Multiple linear regression with the “enter” method was performed to determine the independent factors correlates of the severity of mobile phone addiction; MPAS score was entered as the dependent variable, while its significant demographic correlates in univariate correlation analyses were the independent variables. The significance level was set at 0.05 (two-sided).

## Results

Altogether, 2,523 students have been invited to participate in the study; 2,312 students (928 from Macau, 938 from mainland China, and 446 from Hong Kong) completed the assessments, yielding a participation rate of 91.6%. No significant difference was observed between students who completed and those who did not complete the survey in terms of gender and age.

Table 1 shows the of sociodemographic characteristics of the whole sample and by study sites. Table 2 displays the association between mean MPAS score and demographic characteristics, the Pearson correlation coefficient between actual sleep hours and MPAS score was −0.161 (*p* < 0.001). Table 3 shows the association between the severity of mobile phone addiction and QOL. MPAS score was significantly and negatively associated with all QOL domains after controlling for covariates, that is, those with more excessive mobile phone use were more likely to have poor QOL in all domains. Multiple linear regression analyses revealed that compared to students in mainland China, students in Macao (β = 2.74, *p* < 0.001) and Hong Kong (β = 2.86, *p* < 0.001) were more likely to have excessive mobile phone use (Table 4). Further, higher academic pressure (β = 1.99, *p* < 0.001) and poor academic performance (β = 1.41, *p* < 0.001) were both positively, while male gender (β = −2.99, *p* < 0.001), greater interest in academic major (β = −2.15, *p* < 0.001) and longer sleep duration (β = −1.13, *p* < 0.001) were negatively associated with excessive mobile phone use.

**Table 1:** Socio-demographic characteristics of the participants.

	Macau (*n* = 928)		Hong Kong (*n* = 446)		Mainland China (*n* = 938)		Whole sample (*n* = 2,312)	
	*n*	%	*n*	%	*n*	%	*n*	%
Male	230	24.8	135	30.3	227	24.2	592	25.6
Only child in family	593	63.9	311	69.7	391	41.7	1,295	56.0
Grade								
First	454	48.9	28	6.3	274	29.2	756	32.7
Second	157	16.9	122	27.3	281	30.0	560	24.2
Third	157	16.9	86	19.3	272	29.0	515	22.3
Fourth and above	160	17.2	210	47.1	111	11.8	481	20.8
Nursing students	246	26.5	0	0	232	24.7	478	20.7
Interest in academic major								
Fair or dislike	382	41.2	318	71.3	507	54.1	1,207	52.2
Like	546	58.8	128	28.7	431	45.9	1,105	47.8
Academic pressure								
Little or no pressure	647	69.7	358	80.3	700	74.6	1,705	73.7
High pressure	281	30.3	88	19.7	238	25.4	607	26.3
Academic performance (point)								
85–100	131	14.1	21	4.7	319	34.0	471	20.3
75–84	349	37.6	183	41.0	407	43.4	939	40.6
66–74	287	30.9	180	40.4	154	16.4	621	26.9
<65	161	17.4	62	13.9	58	6.2	281	12.2
Perspective on future								
Fair or pessimistic	502	54.1	390	87.4	553	59.0	1,445	62.5
Optimistic	426	45.9	56	12.6	385	41.0	867	37.5
	Mean	SD	Mean	SD	Mean	SD	Mean	SD
Age (years)	19.7	1.6	21.2	1.6	20.3	1.4	20.3	1.6
MPAS score	33.6	10.6	34.4	10.4	29.7	11.1	32.2	11.0
Actual sleep hours	6.46	1.24	6.46	1.4	6.75	1.04	6.58	1.2

**Note:**

BMI, body mass index; MPAS, mobile phone addiction scale; QOL, quality of life.

**Table 2:** Univariate analyses of factors associated with severity of mobile phone addiction.

Variable	MPAS score statistics		
	Mean ± SD	*t*/*F*	*p*
Gender^a^		4.7	**<0.001**
Male	30.35 ± 11.61		
Female	32.86 ± 10.76		
Location of university^b^		42.0	**<0.001**
Macao	33.64 ± 10.68		
Hong Kong	34.47 ± 10.46		
Mainland China	29.74 ± 11.18		
Only child in family^a^		−5.2	**<0.001**
Yes	33.28 ± 10.64		
No	30.86 ± 11.38		
Grade^b^		2.1	0.08
First	32.25 ± 11.21		
Second	32.13 ± 10.94		
Third	31.38 ± 10.86		
Fourth and above	33.16 ± 11.01		
Nursing students^a^		−0.1	0.8
No	32.19 ± 11.06		
Yes	32.29 ± 10.95		
Interest in academic major^a^		6.1	**<0.001**
Fair or dislike	33.55 ± 10.93		
Like	30.76 ± 10.97		
Academic stress^a^		−5.4	**<0.001**
Little or no stress	31.47 ± 10.81		
High stress	34.30 ± 11.41		
Academic performance (point)^b^		36.8	**<0.001**
85–100	28.37 ± 11.08		
75–84	32.15 ± 10.81		
66–74	33.28 ± 10.64		
<65	36.52 ± 10.52		
Perspective on future^a^		4.3	**<0.001**
Fair or pessimistic	32.98 ± 10.90		
Optimistic	30.94 ± 11.15		

**Notes:**

a Independent-samples *t* test.

b Analysis of Variance (ANOVA) test.

Bolded values are *p* < 0.05.

MPAS, mobile phone addiction scale.

**Table 3:** Association between severity of mobile phone addiction and quality of life.

Variable	MPAS score
Physical QOL^a^	−0.247**
Psychological QOL^a^	−0.251**
Social QOL^a^	−0.184**
Environmental QOL^a^	−0.184**

**Notes:**

** *p* < 0.01.

a After controlling the variables significantly associated with MPAS score in univariate analyses.

MPAS, mobile phone addiction scale; QOL, quality of life.

**Table 4:** Independent correlates of severity of mobile phone addiction.

Variable	*B*	Beta	*t*	*p*
Male gender	−2.99	−0.11	−5.7	**<0.001**
Living in Macao	2.74	0.12	5.1	**<0.001**
Living in Hong Kong	2.86	0.10	4.1	**<0.001**
Actual sleep hours	−1.13	−0.12	−6.1	**<0.001**
Only child in family	0.77	0.035	1.6	0.09
Interested in academic major	−2.15	−0.097	−4.4	**<0.001**
High academic pressure	1.99	0.079	3.8	**<0.001**
Positive perspective on future	−0.58	−0.026	−1.1	0.25
Poor academic performance	1.41	0.11	5.2	**<0.001**

**Note:**

Bolded values are *p* < 0.05.

## Discussion

To the best of our knowledge, this was the first study that examined the demographics and QOL in Chinese university students addicted to the use of mobile phone. The severity of mobile phone addiction was different across study sites. Compared to students in mainland China, their counterparts in Macao and Hong Kong were more likely to suffer from more excessive mobile phone use, which could be attributed to several reasons. First, there is difference in the availability of mobile phones between study sites; Macau has 206.43 mobile phones per 100 people, followed by Hong Kong (190.21 mobile phones per 100 people), while in mainland China the corresponding figure is just 64.04 mobile phones per 100 people (International Telecommunications Union, 2011). Second, due to different socio-economic background between the three locations, free Wi-Fi internet service was available in participating universities in Macau and Hong Kong, but not in those in mainland China, which could partly explain the more excessive mobile phone use in Macao and Hong Kong.

Male students were found less likely to have more excessive mobile phone use in this study, which is consistent with the findings in Korean students (Choi et al., 2015) and in other populations elsewhere (Jenaro et al., 2009; Sanchez-Martinez & Otero, 2009). The more frequent and excessive mobile phone use by female students is probably associated with the different patterns (Hans, 2006; Roberts, Yaya & Manolis, 2014) and purpose of mobile phone use between genders (Choi et al., 2015). Female students usually use mobile phones for conversations, personal messaging, and sending emails to maintain interpersonal relationship and broaden their social network (Bianchi & Phillips, 2005; Bonka, Robert & David, 2001; Fernandez-Villa et al., 2015; Junco, Merson & Salter, 2010; Langer et al., 2017); in contrast, for male students mobile phone is more often an entertainment tool (Hans, 2006). In addition, female students are more likely to use multimedia applications by smartphones (Chen et al., 2017).

Similar to previous findings (Lepp, Barkley & Karpinski, 2015), a positive association between poor academic performance (*B* = 1.41, *p* < 0.001) and excessive mobile phone use was found in this survey. Students with more excessive mobile phone prefer to use more superficial approach to learning (e.g., instrumental learning to meet the requirements of one’s learning outcomes), instead of a deep approach (e.g., to fully understand the content studied), which could result in poor academic performance (Rozgonjuk, Saal & Taht, 2018). Students with greater interest in their academic major were less likely to have excessive mobile phone use (*B* = −2.15, *p* < 0.001). Excessive mobile phone use may lead to concentration difficulties in class and doing homework leading to poor academic performance (Jacobsen & Forste, 2011; Junco & Cotten, 2012). With increased stress, self-control usually decreases, contributing to the risk of excessive mobile phone use (Cho, Kim & Park, 2017); in addition, students with high academic pressure may attempt to relax by using mobile phone frequently, which could further explain the positive association between heavy academic pressure and excessive mobile phone use. A negative association between sleep duration and excessive mobile phone use was also found in this study. All electronic devices could have a negative impact on sleep time and quality (Zarghami et al., 2015). Many people use mobile phone at bedtime and the blue wavelength light from screens could affect the regulation of melatonin, which is associated with shortened sleep time and poor sleep quality (Schuz et al., 2009).

Students with excessive mobile phone use are more likely have unhealthy and irregular life habits and sedentary behaviors and spend less time on physical exercise (Kim, Kim & Jee, 2015), ending up with various health problems, such as obesity or metabolic syndrome (Kautiainen et al., 2005), low back, neck and shoulder pain, blurred vision (Rai et al., 2016), and depression (Thomee, Harenstam & Hagberg, 2011). All these negative health outcomes lower QOL. Excessive mobile phone use had negative associations across all QOL domains in this survey.

The strengths of this study include the large sample size and multicenter design. However, there were also limitations. First, due to the cross-sectional design, causality between the severity of mobile phone addiction and its background variables could not be examined. Second, important pieces of information related to mobile phone use, such as duration and frequency of mobile phone use, time spent on different hobbies, economic status, personality and social support, were not recorded. Due to logistical reasons, participants were only recruited from Beijing and Jilin province, therefore the findings could not be generalized to all university students in mainland China. Third, the instruments were self-reported screening tools and not diagnostic tools. There has been no agreement on the definition and diagnostic criteria of mobile phone addiction, therefore, following other studies (Chiu, Hong & Chiu, 2013; Hong et al., 2019; Lu et al., 2019), only a self-reported scale on the severity of mobile phone addiction could be employed.

## Conclusions

Due to the negative impact of excessive mobile phone use on all aspects of QOL, effective preventive measures, such as public education, should been developed for Chinese university students, especially for those in Macau and Hong Kong.

## Supplemental Information

10.7717/peerj.8859/supp-1 Supplemental Information 1 Data.

10.7717/peerj.8859/supp-2 Supplemental Information 2 Data collection form (English version).

## Abbreviations

CINIC: The China Internet Network Information Center
QOL: Quality of life
MPAS: Mobile Phone Addiction Scale
WHOQOL-BREF: World Health Organization Quality of life—brief version
BMI: Body mass index

## Additional Information and Declarations

### Data Availability

The following information was supplied regarding data availability: The raw measurements are available in the Supplemental Files.
