# Supplementary material for: The severity of mobile phone addiction and its relationship with quality of life in Chinese university students
Source: PeerJ. 2020 Jun 1;8:e8859. doi: 10.7717/peerj.8859 (PMC7271884; doi:10.7717/peerj.8859)
Supplement: Supplemental Information 2 [file peerj-08-8859-s002.doc]

Socio-democratic information

1. Age
2. Sex：□1=male; □2=female
3. Native place：□1=Macau; □2=Hong kong ;□3=Mainland
4. Religion： □0＝No religion; □1＝yes;
5. Single Child: □0＝No; □1＝yes;
6. weight（kg） height（m）
7. Do you have major medical diseases currently? □0＝No; □1＝yes;
8. History of mental health in your family:□1＝no ; □2＝schizophrenia; □3＝Affective disorders; □4＝Neurosis(obsessive compulsive disorder, anxiety disorder); □5＝alcohol or drug abuse and dependence; □6＝other psychological disorder
9. Physical examination

| Item | Result |
| --- | --- |
| weight（kg） |  |
| height（m） |  |
| waistline（cm） |  |

1. College: □1＝University of Macau; □2＝Kiang Wu Nursing College of Macau; □3＝Beijing Union Nursing University; □4＝Beijing Normal University; □5＝Jinlin Unversity
2. What is your major?
3. Year of study : □1=first; □2=second ; □3=third ; □4=forth ; □5= fifth
4. Interest in your major : □1＝like very much; □2＝like; □3＝disliked
5. Do you satisfied with your school? □1＝satisfied; □2＝unsatisfied
6. Do you satisfied with your lifestyle and social life？ □1＝satisfied; □2＝unsatisfied
7. Academic stress: □1＝greater; □2＝medium; □3＝less
8. Do you have difficult in study？ □1＝yes; □2＝no
9. Average score: □1＝85-100 ; □2＝75-84 ; □3＝66-74 ；□4＝65-70; □5＝<60
10. Academic performance: □1＝better；□2＝medium；□3＝worse
11. Perspective toward future career: □1＝good; □2＝fair; □3＝poor
12. Interpersonal relationship: □1＝good; □2＝fair ; □3＝poor
13. Self-reported physical status: □1＝very good；□2＝good；□3＝bad；□4＝poor
14. Self-reported mental health status: □1＝very good；□2＝good；□3＝bad；□4＝poor
15. Marital status of your parents: □1＝normal;  □2＝divorces; □3＝separation；□4＝others ( )
16. How long do you sleep every day in the last month? _______

**Smoking status**

1. Do you smoke?

□1＝No

□2＝I had ever smoked（at least one cigarette per day and at least last once）

□3＝I am smoking （at least one cigarette per day last month）

1. How much cigarette do you smoke every day? ______

| 1. What is your age when you regular smoke? ______ 2. Hve you tried to stop smoking？ □1＝Yes □2＝No |
| --- |

**The instruments in the current study include:**

**MPAS**

1. While using mobile phones, I would think " some more minutes"
2. I have tried to decrease mobile phone usage time, but you have failed
3. While not using the mobile phone, I still think about using the mobile phone and have visions about using the mobile phone.

4) using mobile phone at night influence my sleep

5) I try to hide my mobile phone usage time

6) Mobile phone usage influences my school work

7) I neglect school work to spend more time on mobile phone usage

8) My school performance and concentration are influenced by mobile phone usage

9) Before having to do something I always check the mobile phone to see whether there are missed calls or text messages

10) I find myself wanting to use the mobile phone again

11) When others ask me what I am doing when I use my mobile phone, I become defensive or secretive.

**WHOQOL-BREF**

**Please read each question, assess your feelings, and circle the number on the scale that gives the best answer for you for each question.**

|  | Very poor | Poor | Neither poor or good | Good | Very good |
| --- | --- | --- | --- | --- | --- |
| 1.How would you rate your quality of life? |  |  |  |  |  |
|  | Very dissatisfied | Dissatisfied | Neither dissatisfied or satisfied | Satisfied | Very satisfied |
| 2. How satisfied are you with your health? |  |  |  |  |  |
| The following questions ask about how much you have experienced certain things in the last two weeks. | Not at all | A little | A moderate amount | Very much | An extreme amount |
| 3.To what extent do you feel that physical pain prevents you from doing what you need to do? |  |  |  |  |  |
| 4.How much do you need any medical treatment to function in your daily life? |  |  |  |  |  |
| 5.How much do you enjoy life? |  |  |  |  |  |
| 6.To what extent do you feel your life to be meaningful? |  |  |  |  |  |
|  | Not at all | Slightly | A moderate amount | Very much | Extremely |
| 7.How well are you able to concentrate? |  |  |  |  |  |
| 8.How safe do you feel in your daily life? |  |  |  |  |  |
| 9.How healthy is your physical environment? |  |  |  |  |  |
| The following questions ask about how completely you experience or were able to do certain things in the last two weeks |  |  |  |  |  |
|  | Not at all | A little | A moderate amount | Mostly | Completely |
| 10.Do you have enough energy for everyday life? |  |  |  |  |  |
| 11.Are you able to accept your bodily appearance? |  |  |  |  |  |
| 12.Have you enough money to meet your needs? |  |  |  |  |  |
| 13.How available to you is the information that you need in your day-to-day life? |  |  |  |  |  |
| 14.To what extent do you have the opportunity for leisure activities? |  |  |  |  |  |
|  | Very poor | Poor | Neither poor or well | Well | Very Well |
| 15. How well are you able to get around? |  |  |  |  |  |
| The following questions ask you to say how good or satisfied you have felt about various aspects of your life over the last two weeks. | Very dissatisfied | Dissatisfied | Neither dissatisfied or satisfied | Satisfied | Very satisfied |
| 16. How satisfied are you with your sleep? |  |  |  |  |  |
| 17. How satisfied are you with your ability to perform your daily living activities? |  |  |  |  |  |
| 18. How satisfied are you with your capacity for work? |  |  |  |  |  |
| 19. How satisfied are you with yourself? |  |  |  |  |  |
| 20. How satisfied are you with your personal relationships? |  |  |  |  |  |
| 21. How satisfied are you with your sex life? |  |  |  |  |  |
| 22.How satisfied are you with the support you get from your friends? |  |  |  |  |  |
| 23.How satisfied are you with the conditions of your living place? |  |  |  |  |  |
| 24. How satisfied are you with your access to health services? |  |  |  |  |  |
| 25. How satisfied are you with your mode of transportation? |  |  |  |  |  |
| The follow question refers to how often you have felt or experienced certain things in the last two weeks. | Never | Seldom | Quite often | Very often | Always |
| 26. How often do you have negative feelings, such as blue mood, despair, anxiety, depression? |  |  |  |  |  |
